# Is the “Common Cold” Our Greatest Ally in the Battle Against SARS-CoV-2?

**DOI:** 10.3389/fcimb.2020.605334

**Published:** 2020-12-18

**Authors:** Manu N. Capoor, Fahad S. Ahmed, Andrew McDowell, Ondrej Slaby

**Affiliations:** ^1^ Laboratory of Bacterial Pathogenesis and Immunology, Rockefeller University, New York, NY, United States; ^2^ Executive Office, MMF Systems, Inc., New York, NY, United States; ^3^ Data Science and Engineering, MyMedicalFiles (MMF) Systems, Inc., New York, NY, United States; ^4^ Nutrition Innovation Centre for Food and Health (NICHE), School of Biomedical Sciences, Ulster University, Coleraine, United Kingdom; ^5^ Central European Institute of Technology (CEITEC), Masaryk University, Brno, Czechia; ^6^ Department of Biology, Faculty of Medicine, Masaryk University, Brno, Czechia

**Keywords:** SARS-CoV-2, COVID-19, T-cell, human coronaviruses, immunity, contact tracing, children

## Abstract

The discovery of T-cell responses to SARS-CoV-2 in non-infected individuals indicates cross-reactive immune memory from prior exposure to human coronaviruses (HCoV) that cause the common cold. This raises the possibility that “immunity” could exist within populations at rates that may be higher than serology studies estimate. Besides specialized research labs, however, there is limited ability to measure HCoV CD4+ and CD8+ T-cell responses to SARS-CoV-2 infection, which currently impedes interpretation of any potential correlation between COVID-19 disease pathogenesis and the calibration of pandemic control measures. Given this limited testing ability, an alternative approach would be to exploit the large cohort of currently available data from which statistically significant associations may be generated. This would necessitate the merging of several public databases including patient and contact tracing, which could be created by relevant public health organizations. Including data from both symptomatic and asymptomatic patients in SARS-CoV-2 databases and surveillance systems could provide the necessary information to allow for more informed decisions.

## Introduction—Asymptomatic Infection in Dense Populations

It is most curious that the majority of poor, highly populated countries have seen fewer deaths per million during the current pandemic than most advanced Western nations. In contrast, during the 1918 influenza pandemic, which had a global death rate approaching 3%, less-developed countries saw noticeably higher rates (Spreeuwenberg et al., 2018). The large variation in SARS-CoV-2 deaths per million people between countries, and even states and counties within the U.S., are on the surface confusing and difficult to explain. It seems counterintuitive that countries with both limited health resources and sick, aging populations have reported far fewer mortalities due to SARS-CoV-2 than countries with advanced healthcare resources, relatively healthy populations and greater lock-down measures (Worldometer; Contributors W; Engineering JHUCfSSa, 2020) (**Table 1**).

**Table 1:** Covid-19 Deaths/Million – 40 most populous countries and United States counties.

Country/Territory	Populace July ‘20	Rank	% World July ‘20	Covid-19 Deaths Nov. ‘20	Deaths/Million	Rank	USA County	State	Populace July 2018	Rank	% US Populace July ‘18	Covid-19 Deaths Nov. ‘20	Deaths/ Million	Rank
Spain	46,754,778	30	0.6%	43,131	922.5	1	Bronx	NY	1,432,132	28	0.4%	5,033	3514.3	1
Italy	60,461,826	23	0.8%	50,453	834.5	2	Queens	NY	2,278,906	11	0.7%	7,338	3220.0	2
UK	67,886,011	21	0.9%	55,327	815.0	3	Kings	NY	2,582,830	9	0.8%	7,481	2896.4	3
Argentina	45,741,007	31	0.6%	37,002	808.9	4	New York	NY	1,628,701	21	0.5%	3,219	1976.4	4
Brazil	212,559,417	6	2.7%	169,183	795.9	5	Wayne	MI	1,753,893	19	0.5%	3,154	1798.3	5
Mexico	128,932,753	10	1.7%	101,676	788.6	6	Nassau	NY	1,358,343	29	0.4%	2,240	1649.1	6
USA	331,002,651	3	4.2%	257,072	776.6	7	Middlesex	MA	1,614,714	22	0.5%	2,385	1477.0	7
France	65,273,511	22	0.8%	49,307	755.4	8	Suffolk	NY	1,481,093	26	0.5%	2,032	1372.0	8
Colombia	50,882,891	29	0.7%	35,287	693.5	9	Miami-Dade	FL	2,761,581	7	0.8%	3,766	1363.7	9
Iran	83,783,942	19	1.1%	45,255	540.1	10	Philadelphia	PA	1,584,138	23	0.5%	1,952	1232.2	10
Poland	37,846,611	38	0.5%	13,774	363.9	11	Cook	IL	5,180,493	2	1.6%	6,303	1216.7	11
South Africa	59,734,218	24	0.8%	20,903	349.9	12	Palm Beach	FL	1,485,941	25	0.5%	1,648	1109.1	12
Canada	37,742,154	39	0.5%	11,535	305.6	13	Oakland	MI	1,259,201	33	0.4%	1,310	1040.3	13
Iraq	40,222,493	36	0.5%	11,996	298.2	14	Maricopa	AZ	4,410,824	4	1.3%	3,896	883.3	14
Ukraine	43,851,044	33	0.6%	11,423	260.5	15	Hennepin	MN	1,259,428	32	0.4%	1,080	857.5	15
Russia	145,934,462	9	1.9%	36,192	248.0	16	Broward	FL	1,951,260	17	0.6%	1,626	833.3	16
Germany	84,339,067	17	1.1%	14,277	169.3	17	Clark	NV	2,231,647	13	0.7%	1,690	757.3	17
Turkey	83,992,949	18	1.1%	12,511	149.0	18	Bexar	TX	1,986,049	16	0.6%	1,473	741.7	18
Morocco	36,910,560	40	0.5%	5,396	146.2	19	Los Angeles	CA	10,105,518	1	3.1%	7,438	736.0	19
India	1,380,004,385	2	17.7%	133,738	96.9	20	Harris	TX	4,698,619	3	1.4%	2,965	631.0	20
Philippines	109,581,078	13	1.4%	8,173	74.6	21	Hillsborough	FL	1,436,888	27	0.4%	884	615.2	21
Egypt	102,334,404	14	1.3%	6,548	64.0	22	Cuyahoga	OH	1,243,857	35	0.4%	734	590.1	22
Indonesia	273,523,615	4	3.5%	16,002	58.5	23	Riverside	CA	2,450,758	10	0.7%	1,400	571.3	23
Algeria	43,849,260	34	0.6%	2,294	52.3	24	Dallas	TX	2,637,772	8	0.8%	1,492	565.6	24
Afghanistan	38,928,346	37	0.5%	1,695	43.5	25	Fairfax	VA	1,150,795	38	0.4%	614	533.5	25
Bangladesh	164,689,383	8	2.1%	6,416	39.0	26	San Bernardino	CA	2,171,603	14	0.7%	1,127	519.0	26
Pakistan	220,892,340	5	2.8%	7,696	34.8	27	Franklin	OH	1,310,300	31	0.4%	664	506.8	27
Myanmar	54,409,800	26	0.7%	1,765	32.4	28	Orange	CA	3,185,968	6	1.0%	1,551	486.8	28
Sudan	43,733,762	35	0.6%	1,197	27.4	29	Tarrant	TX	2,084,931	15	0.6%	982	471.0	29
Kenya	53,771,296	27	0.7%	1,392	25.9	30	Orange	FL	1,348,975	30	0.4%	625	463.3	30
Japan	126,476,461	11	1.6%	1,949	15.4	31	Mecklenburg	SC	1,093,901	40	0.3%	429	392.2	31
Ethiopia	114,963,588	12	1.5%	1,651	14.4	32	Allegheny	PA	1,218,452	36	0.4%	475	389.8	32
South Korea	51,269,185	28	0.7%	509	9.9	33	Travis	TX	1,248,743	34	0.4%	476	381.2	33
Nigeria	206,139,589	7	2.6%	1,167	5.7	34	King	WA	2,233,163	12	0.7%	847	379.3	34
Uganda	45,195,774	32	0.6%	172	3.8	35	Sacramento	CA	1,540,975	24	0.5%	546	354.3	35
DR Congo	89,561,403	16	1.1%	329	3.7	36	Salt Lake	UT	1,152,633	37	0.4%	377	327.1	36
China	1,439,323,776	1	18.5%	4,742	3.3	37	Alameda	CA	1,666,753	20	0.5%	499	299.4	37
Thailand	69,799,978	20	0.9%	60	0.9	38	San Diego	CA	3,343,364	5	1.0%	966	288.9	38
Vietnam	97,338,579	15	1.2%	35	0.4	39	Santa Clara	CA	1,937,570	18	0.6%	463	239.0	39
Tanzania	59,308,690	25	0.8%	21	0.4	40	Contra Costa	CA	1,150,215	39	0.4%	257	223.4	40
Totals:	6,448,947,037		82.7%	1,179,251					88,652,927		27.1%	83,437		

While a number of factors may explain this anomaly, including differences in age, co-morbidities, socio-economic status, and testing capacity, it should not be overlooked that individuals in these poorer countries, who often reside in crowded multi-generational family groups with shared facilities, may have experienced increased and possibly sustained, exposure to human coronaviruses (HCoVs) that cause the “common cold”. This elevated exposure, which may lead to greater levels of cross-immunity to SARS-CoV-2, may help explain why:

- A serology study of slums in three wards of Mumbai showed that residents there had 3·5 times the SARS-CoV-2 infection rate of those in housing societies (57% versus 16%), but lower fatality rates (0·05%–0·10%) (Malani et al., 2020). None of the people included in the study had been tested for active SARS-CoV-2 infection using an RT-qPCR assay, suggesting either asymptomatic infection or very mild symptoms.
- A study of the United States state prison systems in Arkansas, North Carolina, Ohio, and Virginia revealed a 70% infection rate amongst the 4,693 inmates tested, of whom 96% were asymptomatic. A significant number of these prisoners were older and, therefore, presumed to be more prone to complications, but this was not the case (So and Smith, 2020).
- Approximately 90% of 147 homeless shelter residents in Boston who were infected with SARS-CoV-2 exhibited no symptoms, even though the shelter’s living space was shared by all residents. Similarly, 95% of the 481 infected workers at a Tyson Foods poultry plant in Springdale, Arkansas were asymptomatic (Cha, 2020).

## Asymptomatic Infection in School Children

Schools are densely populated environments, so their reopening has left many people anxious about the role they may play in spreading SARS-CoV-2 within homes and the wider community. Data has consistently shown that children who become infected with SARS-CoV-2 typically experience mild or no symptoms. There may be multiple reasons for this clinical outcome, including the lack of comorbidities and/or age-related immune system characteristics. However, an intriguing possibility is that cross-reactive T-cells generated in response to HCoV-related colds may be present at a higher prevalence within the younger population due to exposure in school environments (Arroll, 2011; Steinman et al., 2020). It may be possible that these “circulating” cross-reactive T-cells could suppress the development of clinical symptoms, despite a recent study demonstrating that viral nucleic acid loads in 5- to 17-year-olds match or exceed (for those less than 5 years old) those found in adults (Heald-Sargent et al., 2020). It is also unclear to what extent infected children shed SARS-CoV-2 or are vectors for its spread, especially those who are symptom-free. Studies to date have suggested that children, especially those in primary or elementary schools, are at low risk of contracting the disease, may be the initial source of infection in only a very small number of cases, and are unlikely to pass it on *via* child-to-child or child-to-adult transmission (Armann et al., 2020, Davies et al., 2020; Munro and Faust, 2020; Posfay-Barbe et al., 2020).

## T-Cell Responses to SARS-CoV-2 in Non-Infected and Asymptomatic Individuals

While T-cell responses to SARS-CoV-2 in infected individuals have now been reported, the emerging descriptions of pre-existing SARS-CoV-2 cross-reactive CD4^+^ and CD8^+^ T-cells amongst individuals who have not been infected are of particular interest (Corman et al., 2018; Braun et al., 2020; Grifoni et al., 2020; Sette and Crotty, 2020). The presence of these cross-reactive T-cells appears to be explained by prior exposure to HCoVs, such as HCoV-HKU1, HCoV-OC43H, CoV-NL63, and HCoV-229E, that generate T-cells capable of recognizing SARS-CoV-2 antigens (Mateus et al., 2020). Though involving different viruses, a similar situation is reminiscent in the historical findings of physician Edward Jenner, who discovered that milkmaids rarely succumbed to smallpox and correctly deduced that exposure to cowpox, a related virus causing a much milder illness, protected them. Similarly, during the 2009 swine flu pandemic caused by H1N1/09, the majority of people over the age of 60 displayed levels of pre-existing immunity resulting from exposure to similar flu viruses earlier in life, while young individuals had very little immunity and were, in contrast to the current situation, at greater risk (Xu et al., 2010).

Immunocompetent children and adults with respiratory infections (the common cold) often present as asymptomatic, or develop mild respiratory tract illnesses. More than 200 viral strains have been implicated as causal factors of the common cold. Rhinoviruses are responsible for ~35% of upper respiratory infections; HCoVs, often asymptomatic, cause 15-25% of cases, while influenza, adenoviruses, and other viral types account for the rest. Adults average two to three common colds each year, while children get six to eight, of which one or two are caused by HCoVs (Simasek and Blandino, 2007). Accordingly, it was found in a study of 100 subjects that most individuals had evidence of serum antibodies to all four HCoV strains (Gorse et al., 2010). We envisage one potential study resulting from a synthesis of these observations could be to investigate whether levels of longer-lasting “immunity” to SARS-CoV-2 could be higher than serology tests indicate. A positive result would be welcome news from a public health perspective.

Our ability to measure HCoV CD4+ and CD8+ T-cell responses to SARS-CoV-2 infection is limited due to the specialized nature of the tests. This major knowledge gap makes the interpretation of COVID-19 disease pathogenesis more difficult and clouds our ability to quantify the usefulness of pandemic control measures, such as social distancing.

## Current Databases Do Not Capture Severity of Symptoms and Cohabitation Data

Searches of relevant publicly available databases, including the Johns Hopkins Coronavirus Resource Centre, National Institutes of Health Open-Access Data and Computational Resources to Address COVID-19, CDC World Dataset, The Covid-19 Tracking Project, Florida Department of Health, New York City Department of Health and Mental Hygiene, 1Point3Acres, and others, revealed a lack of information relating to the severity of symptoms (symptomatic vs. asymptomatic), possibly related to lower testing rates in highly populated or developing countries for individuals who are asymptomatic. More importantly, information pertaining to cohabitation and contact tracing was not included. A retrospective analysis that combines information from SARS-CoV-2 patient and contact tracing databases might be useful in determining whether individuals infected with SARS-CoV-2 who reside with school-age children are more likely to be asymptomatic. Such an analysis could be used to test the hypothesis that exposure to HCoV in densely populated environments leads to greater levels of cross-immunity to SARS-CoV-2.

## The Need to Include Asymptomatic Individuals in Surveillance Studies

Surveillance studies of respiratory virus circulation in the United States (and globally) are typically limited to data collected by clinical labs from symptomatic individuals. This approach does not capture the impact of respiratory infections that are asymptomatic or associated with mild respiratory tract illness in immunocompetent children and adults. The largest such surveillance study of symptomatic HCoV patients, which was conducted in the U.S. from 1^st^ July, 2014 through 30^th^ June, 2017, contained the results of 854,575 HCoV rRT-PCR tests performed by 117 laboratories in 42 states. Overall, 2·2% were positive for HCoV-OC43, 1·0% for HCoV-NL63, 0·8% for HCoV-229E, and 0·6% for HCoV-HKU1 (Killerby et al., 2018). These data suggest that less than about 5% of symptomatic HCoV infections (colds) are likely to help build cross-immunity to SARS-CoV-2.

For a different perspective we turn to a unique, cross-sectional longitudinal study of individuals, both symptomatic and asymptomatic, that sought to identify changes in respiratory virus colonization before and after the 2013 Hajj, a 5- to 6-day religious pilgrimage to Mecca, Saudi Arabia (attended by more than 2 million people) (Memish et al., 2015). Pre- and post-Hajj nasal specimens were prospectively obtained from a paired cohort (692 pilgrims) and from nonpaired cohorts (514 arriving and 470 departing pilgrims) from 13 countries including the United States (8·4%) and countries in Africa (44·2%), Asia (40·2%), and Europe (7·2%). Nasal specimens were tested for 34 respiratory pathogens using RT-PCR. The prevalence of viruses increased from 7·4% before the Hajj to 45·4% after the Hajj, due to the acquisition of rhinoviruses, coronaviruses (229E, HKU1, OC43), and influenza A H1N1. Among the paired cohort, HCoV 229E infections increased from six to 101 (0·9%–14·6%; *p* < 0·001). Among the non-paired cohort, HCoV 229E infections increased from five to 48 (1·0%–10·2%; *p* < 0·001). These data, taken from both symptomatic and asymptomatic adults, show that HCoV 229E can, given the right conditions, spread extremely quickly in a short period of time. This supports the hypothesis that children, and potentially the adults with whom they live, are likely to have been exposed to HCoV strains that can elicit T-cells cross-reactive to SARS-CoV-2, perhaps leading to a clinically relevant easing of symptoms. However, acquiring the information needed to truly test the hypothesis, and make more informed decisions, necessitates the inclusion of data from both asymptomatic and symptomatic patients, SARS-CoV-2 databases, and surveillance systems.

## Conclusion

It may be the case that seasonal HCoV colds have actually been, and will continue to be, one of our greatest allies in the fight against the current SARS-CoV-2 pandemic and possibly SARS-CoV-1 which surfaced in November 2002 (Feng et al., 2009).

Given other HCoVs are likely to provide levels of protection against SARS-CoV-2 *via* cross-reactive immune memory, it is important to maintain levels of immunity to the broader spectrum of HCoVs in the current environment. If the spread of other mild respiratory and HCoV-related infections in school is reduced from normal levels, what impact will this have on the normal transmission of the “common cold” between children and those with whom they reside, as well as the wider community? Could this lead to a reduction in the temporary buildup of cross-reactive T-cell responses to SARS-CoV-2 that could provide some vital protection in an older, higher risk population, particularly given the reduced level of T-cell diversity and loss of immunity to previous HCoV infections as individuals age? While speculative at this stage, it is possible that differential spread of respiratory viruses other than SARS-CoV-2 within the younger population could be beneficial for their older relatives.

Besides specialized research labs there is a limited ability to measure HCoV CD4+ and CD8+ T-cell responses to SARS-CoV-2 infection. This major knowledge gap currently impedes interpretation of COVID-19 disease pathogenesis, and the calibration of pandemic control measures including social distancing. Unfortunately, the data needed to demonstrate this is not available because current SARS-CoV-2 databases and surveillance systems do not include information about the people who might be benefitting from cross-reactive immune memory ie., those who are asymptomatic. Inclusion of this data could help paint a more accurate picture of the clinical course of the SARS-CoV-2 pandemic and enable better decision making.

## Data Availability Statement

The original contributions presented in the study are included in the article/supplementary material. Further inquiries can be directed to the corresponding author.

## Author Contributions

MC conceived the idea for the manuscript and wrote the first draft. MC, FA, AM, and OS participated in rewriting, editing, and conducting literature search. All authors contributed to the article and approved the submitted version.

## Conflict of Interest

Authors MC and FA were employed by the company MMF Systems, Inc.

The remaining authors declare that the research was conducted in the absence of any commercial or financial relationships that could be construed as a potential conflict of interest.
